# Comparing the effectiveness and cost-effectiveness of sulfonylureas and newer diabetes drugs as second-line therapy for patients with type 2 diabetes

**DOI:** 10.1136/bmjdrc-2023-003991

**Published:** 2024-05-27

**Authors:** Matteo Franchi, Giacomo Pellegrini, Angelo Avogaro, Giuliano Buzzetti, Riccardo Candido, Arturo Cavaliere, Agostino Consoli, Irene Marzona, Francesco Saverio Mennini, Stefano Palcic, Giovanni Corrao

**Affiliations:** 1 Unit of Biostatistics, Epidemiology and Public Health, Department of Statistics and Quantitative Methods, University of Milano-Bicocca, Milan, Italy; 2 National Centre for Healthcare Research and Pharmacoepidemiology, University of Milano-Bicocca, Milan, Italy; 3 Department of Medicine, Division of Metabolic Diseases, University of Padova, Padua, Italy; 4 Dephaforum, Milan, Italy; 5 Department of Medical, Surgical and Health Sciences, University of Trieste, Trieste, Italy; 6 SC Patologie Diabetiche, ASUGI, Trieste, Italy; 7 Farmaceutica Aziendale, ASL 9 Viterbo, Viterbo, Italy; 8 DMSI and CAST, Università degli Studi Gabriele d'Annunzio Chieti-Pescara, Chieti, Italy; 9 Centre for Economics and International Studies–Economic Evaluation and Health Technology Assessment, Università degli Studi di Roma Tor Vergata, Roma, Italy; 10 SC Farmacia Ospedaliera e Territoriale-Area Giuliana, ASUGI, Trieste, Italy

**Keywords:** Diabetes Mellitus, Type 2, Hypoglycemic Agents, Health Care Costs, Pharmacoepidemiology

## Abstract

**Introduction:**

We aimed to compare the effectiveness and cost-effectiveness profiles of glucagon-like peptide-1 receptor agonist (GLP-1-RA), sodium-glucose cotransporter 2 inhibitor (SGLT2i), and dipeptidyl peptidase-4 inhibitor (DPP-4i) compared with sulfonylureas and glinides (SU).

**Research design and methods:**

Population-based retrospective cohort study based on linked regional healthcare utilization databases. The cohort included all residents in Lombardy aged ≥40 years, treated with metformin in 2014, who started a second-line treatment between 2015 and 2018 with SU, GLP-1-RA, SGLT2i, or DPP-4i. For each cohort member who started SU, one patient who began other second-line treatments was randomly selected and matched for sex, age, Multisource Comorbidity Score, and previous duration of metformin treatment. Cohort members were followed up until December 31, 2022. The association between second-line treatment and clinical outcomes was assessed using Cox proportional hazards models. The incremental cost-effectiveness ratios (ICERs) were calculated and compared between newer diabetes drugs and SU.

**Results:**

Overall, 22 867 patients with diabetes were included in the cohort, among which 10 577, 8125, 2893 and 1272 started a second-line treatment with SU, DPP-4i, SGLT2i and GLP-1-RA, respectively. Among these, 1208 patients for each group were included in the matched cohort. As compared with SU, those treated with DPP-4i, SGLT2i and GLP-1-RA were associated to a risk reduction for hospitalization for major adverse cardiovascular events (MACE) of 22% (95% CI 3% to 37%), 29% (95% CI 12% to 44%) and 41% (95% CI 26% to 53%), respectively. The ICER values indicated an average gain of €96.2 and €75.7 each month free from MACE for patients on DPP-4i and SGLT2i, respectively.

**Conclusions:**

Newer diabetes drugs are more effective and cost-effective second-line options for the treatment of type 2 diabetes than SUs.

**WHAT IS ALREADY KNOWN ON THIS TOPIC**

- Several studies have shown that newer glucose-lowering agents, including glucagon-like peptide-1 receptor agonist (GLP-1-RA), sodium-glucose cotransporter 2 inhibitor (SGLT2i), and dipeptidyl peptidase-4 inhibitor (DPP-4i), are more cost-effective than conventional antidiabetic medications. However, these studies were not explicitly designed to evaluate second-line strategies, and they often used model-based simulations using data derived from clinical trials or literature.

**WHAT THIS STUDY ADDS**

- Based on the observation of a large, unselected population of patients with type 2 diabetes starting second-line therapy after metformin monotherapy failure, it was observed that newer antidiabetic agents were more effective than sulfonylureas and glinides in delaying cardiovascular events and death. Moreover, SGLT2i and DPP-4i were also cost-effective.

**HOW THIS STUDY MIGHT AFFECT RESEARCH, PRACTICE OR POLICY**

- Personalizing the choice of newer second-line agents to individual characteristics and risk profiles may be critical for regulators, clinicians, and patients.

## Introduction

 Pharmacologic treatment of type 2 diabetes, along with comprehensive lifestyle modification, typically starts with metformin, unless contraindicated.^1^ The addition of diabetes drugs acting by mechanisms different from metformin (second-line therapy) is recommended whenever metformin fails to achieve acceptable glucose levels.^2^ Failure of metformin to achieve optimal glycemic control is common in clinical practice.^3^

Several second-line drugs are available, including conventional drugs such as sulfonylureas or glinides (SU), and newer agents for diabetes treatment such as dipeptidyl peptidase-4 inhibitors (DPP-4i), sodium-glucose cotransporter 2 inhibitors (SGLT2i), and glucagon-like peptide-1 receptor agonists (GLP-1-RA).^4^ Conventional second-line therapy might lead to a reasonably acceptable glycemic control, although associated with an increased risk of hypoglycemia; therefore, sulfonylureas and metformin have long been regarded as the most cost-effective second-line therapy following failure of diet and exercise intervention.^5^ However, increasing evidence suggests that newer glucose-lowering agents can reduce the risk of cardiovascular (CV) events and mortality in patients at high CV risk.^6 7^ These new medications are steadily replacing SU as the most common treatment in patients for whom metformin has failed.^8^ Consequently, this shift to newer glucose-lowering agents has caused increased treatment costs, which could be outweighed from savings deriving from the CV benefits associated with their use.^9^

Several studies have shown that newer glucose-lowering agents, including GLP-1-RA, SGLT2i, and DPP-4i, are more cost-effective than conventional diabetes medications.^10 11^ However, these studies were not explicitly designed to evaluate second-line strategies: moreover, they often used model-based simulations using data derived from clinical trials or literature.^12^ Only one study has been based on real-world data.^13^ These approaches limit the generalizability of results; thus, further comparative investigations are needed to understand the effectiveness and cost-effectiveness of different strategies for second-line treatment of type 2 diabetes in clinical practice.

To address this issue, we performed a large population-based retrospective cohort study to compare the effectiveness and cost-effectiveness profiles of GLP-1-RA, SGLT2i, and DPP-4i relative to SU, in the real world of Italy. Controlling for sources of systematic uncertainty was of particular concern in this study.

## Materials and methods

### Target population and data sources

Residents in Lombardy aged 40 years or older who were beneficiaries of the Regional Health Service formed the main target population (just over 6 million people, approximately 17% of the Italian population in that age group). Italian citizens have equal access to essential healthcare services provided by the National Health Service (NHS). In Lombardy, this has been coupled with an automated system of databases created to collect a variety of information, including (1) an archive of residents who receive NHS assistance (the whole resident population), reporting demographic and administrative data; (2) a database on diagnosis at discharge from public or private hospitals of the Region; (3) a database on outpatient drug prescriptions reimbursed by the NHS; and (4) a database on provision of outpatient visits, including visits in specialist ambulatories and diagnostic laboratories accredited by the NHS (this database contains, among others, the date in which the visit was carried out, the identification code of the visit and the cost incurred by the NHS, but does not contain information on clinical parameters). These various types of data can be interconnected, since all databases use a single individual identification code for each citizen enrolled. To preserve privacy, each identification code was automatically deidentified; the inverse process was only allowed to the Regional Health Authority on request from judicial authorities.

Details of regional databases and their use for studies, including patients with type 2 diabetes, are reported elsewhere.^14^ Online supplemental table S1 reports the list of International Classification of Diseases, 9th Revision with Clinical Modification and Anatomical Therapeutic Chemical codes used in the current study.

### Cohort selection and measurements

All citizens belonging to the target population treated with metformin on December 31, 2014 were eligible for inclusion in this study. This included Lombardy citizens who had at least three prescriptions of metformin dispensed during 2014 with at least one prescription dispensed during December 2014. Patients who were beneficiaries of the regional health service for less than 2 years as of December 31, 2014, and previously received any diabetes treatment, including SU, GLP-1-RA, SGLT2i, DPP-4i, or insulin, were excluded.

Patients who started a second-line treatment between January 1, 2015 and December 31, 2018 were identified. Second-line therapy included adding SU, GLP-1-RA, SGLT2i, or DPP-4i, or replacing metformin with these agents. The date on which the second-line therapy was initiated was considered the index date.

Baseline features measured at the index date included sex, age, duration of metformin treatment in years, and selected co-treatments and comorbidities. Co-treatments were tracked in the 2 years before the index date using previous prescriptions of antihypertensive, antiplatelet, anticoagulant, antidepressant, respiratory, and non-steroidal anti-inflammatory drugs. Comorbidities were tracked in the 2 years before the index date using inpatient diagnosis, including stroke, heart failure, myocardial infarction, renal, respiratory, and neurological diseases, retinopathy, cancer, and depression. In addition, patients were categorized according to the Multisource Comorbidity Score (MCS), a new index of clinical complexity derived from inpatient diagnostic information and outpatient drug prescriptions provided by regional Italian data and validated for outcome prediction.^15^

### Matching design and follow-up

To compare SU with each of the newer second-line agents, a 1:1:1:1 matching design was used. A risk set comprised four individually paired patients who differed as related to their second-line drug therapy but were other ways balanced for several factors. For each cohort member who started SU as a second-line therapy, one patient was randomly selected from the study cohort among those who began other second-line treatments and matched for sex, age, and MCS. Patients included in risk sets were considered to belong to the main study cohort.

Cohort members accumulated person-years (PY) of follow-up, starting from the index date until the earliest date of occurrence of clinical outcome (see below), the administrative censoring for either death or migration to another region, or December 31, 2022. Additional censoring criteria were used for controlling exposure misclassification (see the Statistical analysis section).

### Clinical and economic outcomes

The occurrence of all-cause death or the earliest occurrence of all-cause death or hospital admission for myocardial infarction, heart failure, or stroke (major adverse cardiovascular events, MACE)^16^ were the primary clinical outcomes of interest. In addition, secondary clinical outcomes were death from CV disease, hospital admission for uncontrolled diabetes, long-term diabetes complications, myocardial infarction, stroke, heart failure, kidney disease, and diabetic nephropathy.

The economic outcome was average per capita cumulative healthcare costs directly sustained by the regional health service for treating patients included in the study cohort during follow-up. Costs were calculated from the amount that the Regional Health Authority reimbursed to health providers and included hospitalizations, dispensed drugs (distinguishing between diabetes agents and free-of-charge other medicines), and outpatient services (distinguishing between those for diabetes monitoring and all other services provided free of charge by regional health services such as specialist visits, laboratory examinations, and instrumental examinations).

### Statistical analysis

The baseline demographic and clinical features of cohort members according to dispensed second-line agents were compared using parametric and non-parametric tests where appropriate.

Cox proportional hazards models were used to estimate HR and 95% CI for risk of clinical outcomes among patients prescribed GLP-1-RA, SGLT2i, or DPP-4i drugs compared with those who received SU. Differences between HRs for diabetes drug pairs were determined using the Bonferroni multiple comparisons test. Analyses were further restricted to the subgroup of patients treated with SU, and outcomes were separately analyzed among second and third-generation sulfonylureas users and glinide users.

Incremental cost-effectiveness ratios (ICERs) were calculated and compared between cohort members who received newer diabetes agents and those who received SU. The ICER was expressed in terms of healthcare cost savings expected by gaining 1 month free from MACE due to the difference in second-line therapeutic strategy.^17^ The cumulative difference in per capita healthcare cost was used as the ICER numerator. The approach from Bang and Tsiatis, which accounts for censored data, was used to calculate per capita cumulative costs.^18^ The restricted mean survival time (RMST), calculated using the area under the Kaplan-Meier curve, represents the average time free from clinical outcomes experienced by each cohort member.^19^ The difference in RMST was used as the ICER denominator. A non-parametric bootstrap method based on 1000 iterations^20^ was used to explore the uncertainty of the cost-effectiveness estimates.^21^

### Sensitivity analyses

Several prespecified sensitivity analyses were performed to test the robustness of results. First, because baseline matching criteria were restricted to selected variables (sex, age, MCS, and years since starting metformin before the index date), the primary analysis may be affected by residual confounding. We used a semiautomated data-adaptive ‘high‐dimensional propensity score (HdPS)’ approach to reduce the potential for confounding by indication.^22^ We included four data dimensions in the algorithm. Within each data dimension, we selected the top 50 most prevalent covariates, including diagnostic, procedural, or dispensed medications, along with the following predefined covariates which were forced into the model: (1) demographics; (2) measures of intensity of healthcare utilization; and (3) risk factors or medications associated with the increased risk of the considered outcomes. Then, we selected the top 200 empirical covariates to be included in the propensity score (PS) estimation. We used a logistic regression model to calculate PS, defined as an individual’s predicted probability of receiving a newer hypoglycemic agent versus SU. We then repeated PS matching as described above.

Second, to account for changes in therapeutic strategy during follow-up, such as switching to or adding insulin therapy or switching from one second-line treatment to another, the follow-up was censored when the therapeutic strategy changed. However, informative censoring may have biased estimates because the reasons for evolving therapeutic strategy may have been related to outcome occurrence. To avoid this bias, an inverse probability-of-censoring weights (IPCW) approach was used.^23^ A weight inversely proportional to the probability of censoring was applied to each observation, and the weight was quantified using a time-dependent Cox regression model. Separate models were generated for patients who received conventional therapies and for those who received newer second-line agents. The censoring weights were stabilized using the probability of censoring, conditional on second-line agents received. The stabilized weights were used to estimate the marginal Cox proportional hazards model parameters for assessing the exposure–outcome association using HR and the corresponding 95% CI. The estimates obtained using the above procedure were compared with those obtained from the unweighted estimates.

Third, because the matching design selectively included patients who received second-line diabetes agents, which yielded reduced statistical power, an unmatched design that included all cohort members who started second-line therapy was used. The Cox regression model for this analysis included variables already used for matching (ie, sex, age, MCS, and years since starting metformin previously the index date) as covariates. Because this approach allowed for increased statistical power, the association between therapeutic strategy and the occurrence of secondary clinical outcomes (see above) was separately estimated.

Fourth, we used a different definition of MACE as a composite outcome including myocardial infarction, stroke and CV deaths.

Fifth, the association between second-line treatments and both primary and secondary clinical outcomes was estimated by using the second-line users of second and third-generation sulfonylureas as comparison groups (excluding, thus, the glinide users from the comparison group as in the main analysis).

Finally, to verify the generalizability of our main findings, the effectiveness profile was estimated, other than for Lombardy (the most populous region of Italy) as described above, using Sicily (the largest region of Italy) as the target population. Despite the expected between-region differences in comorbidity profile,^15^ physician behavior, and healthcare policies, the invariance of the impact of second-line agents on the occurrence of clinical outcomes in the real world has been investigated to verify our findings’ generalizability.

All analyses were performed using SAS V.9.4. A two-sided p value of 0.05 or less was considered significant.

## Results

### Patients

The distribution of the exclusion criteria is shown in online supplemental figure S1. Among the 110 073 metformin users on January 1, 2015, a total of 22 867 started second-line therapy for 497 909 person-months (PMs) at risk, with an incidence rate of 45.9 patients with type 2 diabetes changing therapeutic strategy every 1000 PMs. Most patients added (or switched to) SU (10 577 patients, incidence rate 21.2 every 1000 PMs), followed by DPP-4i (8125 patients, incidence rate 16.3 every 1000 PMs), SGLT2i (2823 patients, incidence rate 5.7 every 1000 PMs), and GLP-1-RA (1272 patients, incidence rate 2.6 every 1000 PMs).

Table 1 shows that the second-line therapeutic strategy was associated with relevant and statistically significant differences in demographic and clinical profiles in the unmatched cohorts. Patients prescribed SU were older, on average, had more severe clinical profiles, and were on metformin for a longer time than those who started second-line therapies with newer agents. Conversely, no substantial differences were observed for the profiles of the matched cohorts. At the index date, 42% of patients were from 60 to 69 years of age (1.2% were 80 or older), 57% were men, 73% had good clinical profile (2% had severe clinical profile), and 48% had started metformin from 5 to 9 years before (20% were on metformin treatment for more than 10 years). Aside from matching variables, no significant differences were observed between groups concerning co-treatments.

**Table 1:** Baseline characteristics of unmatched and matched cohorts

	Unmatched cohort, n (%)				Matched cohort, n (%)			
	SU n=10 577	DPP-4i n=8125	SGLT2i n=2893	GLP-1-RA n=1272	SU n=1208	DPP-4i n=1208	SGLT2i n=1208	GLP-1-RA n=1208
Median follow-up (months)	67.6	67.2	64.5	66.9	74.6	72.5	66.8	65.3
Males	5769 (54.5)	4764 (58.6)	1877 (64.9)	718 (56.5)	686 (56.8)	686 (56.8)	686 (56.8)	686 (56.8)
Age categories								
<60	1783 (16.9)	1415 (17.4)	986 (34.1)	507 (39.9)	465 (38.5)	458 (37.9)	462 (38.3)	462 (38.3)
60–69	2786 (26.3)	2415 (29.7)	1237 (42.8)	520 (40.9)	504 (41.7)	516 (42.7)	509 (42.1)	508 (42.1)
70–79	3742 (35.4)	2903 (35.7)	616 (21.3)	229 (18.0)	224 (18.5)	219 (18.1)	224 (18.5)	224 (18.5)
≥80	2266 (21.4)	1392 (17.1)	54 (1.9)	16 (1.3)	15 (1.2)	15 (1.2)	13 (1.1)	14 (1.2)
Duration of treatment with metformin at index date (years)								
<5	3155 (29.8)	2160 (26.6)	884 (30.6)	451 (35.5)	422 (34.9)	384 (31.8)	421 (34.9)	422 (34.9)
5–9	4934 (46.7)	3953 (48.7)	1439 (49.7)	572 (45.0)	558 (48.2)	579 (47.9)	575 (47.6)	549 (45.5)
≥10	2488 (23.5)	2012 (24.8)	570 (19.7)	249 (19.6)	228 (18.9)	245 (20.3)	212 (17.6)	237 (19.6)
Co-treatments								
Antihypertensive	8799 (83.2)	6682 (82.2)	2368 (81.9)	1055 (82.9)	944 (78.2)	950 (78.6)	979 (81.0)	999 (82.7)
Antiplatelet	3959 (37.4)	3064 (37.7)	947 (32.7)	374 (29.4)	363 (30.1)	375 (31.0)	345 (28.6)	359 (29.7)
Anticoagulant	1088 (10.3)	803 (9.9)	132 (4.5)	55 (4.3)	51 (4.2)	54 (4.5)	51 (4.2)	51 (4.2)
Antidepressant	1808 (17.1)	1014 (12.5)	303 (10.5)	171 (13.4)	128 (10.6)	133 (11.0)	140 (11.6)	152 (12.6)
Respiratory drugs	2544 (24.1)	1772 (21.8)	577 (19.9)	293 (23.0)	249 (20.6)	252 (20.9)	269 (22.6)	273 (20.9)
NSAIDs	4005 (37.9)	2860 (35.2)	989 (34.2)	444 (34.9)	422 (34.9)	399 (33.0)	420 (34.8)	422 (34.9)
Comorbidities*								
Stroke	253 (2.4)	160 (2.0)	29 (1.0)	11 (0.9)	17 (1.4)	14 (1.2)	9 (0.8)	11 (0.9)
Heart failure	624 (5.9)	331 (4.1)	47 (1.6)	16 (1.3)	21 (1.7)	20 (1.7)	15 (1.2)	15 (1.2)
Myocardial infarction	308 (2.9)	274 (3.4)	94 (3.3)	21 (1.7)	24 (2.0)	32 (2.7)	14 (1.2)	19 (1.6)
Renal diseases	289 (2.7)	157 (1.9)	4 (0.1)	7 (0.6)	5 (0.4)	4 (0.3)	3 (0.3)	2 (0.2)
Respiratory diseases	794 (7.5)	348 (4.3)	57 (2.0)	35 (2.8)	37 (3.1)	30 (2.5)	26 (2.2)	31 (2.6)
Neurological diseases	44 (0.4)	38 (0.5)	10 (0.4)	2 (0.2)	1 (0.1)	4 (0.3)	0 (0.1)	1 (0.1)
Retinopathy	13 (0.1)	6 (0.1)	2 (0.1)	0 (0.0)	1 (0.1)	0 (0.0)	0 (0.0)	0 (0.0)
Cancer	684 (6.5)	330 (4.1)	68 (2.4)	38 (3.0)	34 (2.8)	33 (2.7)	34 (2.8)	36 (3.0)
Depression	37 (0.4)	7 (0.1)	6 (0.2)	1 (0.1)	3 (0.3)	0 (0.0)	0 (0.0)	0 (0.0)
Multisource Comorbidity Score								
Low	5722 (54.1)	4908 (60.4)	2108 (72.9)	887 (69.7)	892 (73.8)	889 (73.6)	879 (72.8)	844 (69.9)
Intermediate	3914 (37.0)	2811 (34.6)	748 (25.9)	355 (27.9)	271 (22.7)	293 (24.3)	310 (25.7)	341 (28.2)
High	941 (8.9)	406 (5.0)	37 (1.3)	30 (2.4)	42 (3.5)	26 (2.2)	19 (1.6)	23 (1.9)

* Tracked using inpatient diagnosis.

DPP-4i, dipeptidyl peptidase-4 inhibitor; GLP-1-RA, glucagon-like peptide-1 receptor agonist; NSAID, non-steroidal anti-inflammatory drug; SGLT2i, sodium-glucose cotransporter 2 inhibitor; SU, sulfonylurea and glinides.

The comparison of baseline characteristics and clinical outcomes of SU users, stratified among second and third-generation sulfonylureas users and glinide users, is shown in online supplemental table S2, according to which it was observed that glinide users were older and had a worse and more severe clinical profile (online supplemental table S2).

### Comparative effectiveness

Table 2 reports the primary and secondary findings for the relationship between baseline exposure to conventional or newer diabetes second-line agents and risk of MACE and all-cause mortality. After mean follow-up ranging from 65.3 months (SGLT2i) to 74.6 months (SU), 234, 176, 157 and 153 MACEs occurred among patients who received SU, DPP-4i, SGLT2i, and GLP-1-RA, respectively, and the corresponding incidence rates were 31.2, 24.1, 23.9, and 22.8 events every 1000 PYs, respectively. Patients who received DPP-4i, SGLT2i, and GLP-1-RA had 22% (95% CI 3% to 37%), 29% (95% CI 12% to 44%), and 41% (95% CI 26% to 53%) respective reductions in risk of MACE relative to those who received SU at baseline. The results showed that GLP-1-RA offered significantly better protection than DPP-4i against MACE (p=0.0269) and better protection than DPP-4i and SGLT2i against all-cause mortality (p=0.0166 and 0.0453).

**Table 2:** Association between second-line therapies and primary clinical outcomes according to the main analysis and selected sensitivity analyses

	Second-line agent			
	SU	DPP-4i	SGLT2i	GLP-1-RA
**Main analysis**				
Patients, n	1208	1208	1208	1208
MACE*				
Events, n (%)	234 (20.2)	176 (15.3)	157 (13.4)	153 (13.1)
HR (95% CI)	Reference	0.78 (0.63 to 0.97)	0.71 (0.56 to 0.88)	0.59 (0.47 to 0.74)
All-cause death				
Events, n (%)	144 (11.9)	101 (8.4)	74 (6.1)	66 (5.5)
HR (95% CI)	Reference	0.73 (0.55 to 0.98)	0.66 (0.49 to 0.89)	0.47 (0.34 to 0.66)
**High-dimensional propensity score approach**				
Patients, n	1095	1095	1095	1095
MACE*				
Events, n (%)	226 (20.6)	197 (18.0)	143 (13.1)	150 (13.7)
HR (95% CI)	Reference	0.86 (0.70 to 1.05)	0.69 (0.55 to 0.86)	0.64 (0.51 to 0.79)
All-cause death				
Events, n (%)	122 (11.1)	95 (8.7)	74 (6.8)	57 (5.2)
HR (95% CI)	Reference	0.77 (0.58 to 1.02)	0.67 (0.49 to 0.91)	0.40 (0.28 to 0.57)
**Probability-of-censoring weights approach**				
Patients, n	10 577	8125	2893	1272
MACE*				
Events, n (%)	713 (6.8)	418 (5.2)	109 (3.8)	47 (3.7)
HR (95% CI)	Reference	0.72 (0.59 to 0.89)	0.59 (0.41 to 0.85)	0.64 (0.39 to 1.04)
All-cause death				
Events, n (%)	1811 (17.1)	829 (10.2)	97 (3.4)	40 (3.2)
HR (95% CI)	Reference	0.75 (0.67 to 0.85)	0.62 (0.47 to 0.82)	0.61 (0.41 to 0.90)
**Different definition of MACE** †				
Patients, n	1208	1208	1208	1208
MACE†				
Events, n (%)	103 (8.9)	88 (7.7)	80 (6.8)	68 (5.8)
HR (95% CI)	Reference	0.80 (0.60 to 1.06)	0.79 (0.59 to 1.06)	0.62 (0.45 to 0.84)
**Changing the target population**				
Patients, n	541	541	541	541
MACE*				
Events, n (%)	60 (11.1)	44 (8.1)	29 (5.4)	45 (8.3)
HR (95% CI)	Reference	0.76 (0.51 to 1.12)	0.52 (0.34 to 0.81)	0.75 (0.51 to 1.11)
All-cause death				
Events, n (%)	32 (5.9)	22 (4.1)	24 (4.4)	21 (3.9)
HR (95% CI)	Reference	0.70 (0.40 to 1.21)	0.28 (0.13 to 0.61)	0.54 (0.30 to 0.98)

* Composite outcome including myocardial infarction, stroke, heart failure or all-cause deaths.

† Composite outcome including myocardial infarction, stoke or cardiovascular (CV) deaths.

DPP-4i, dipeptidyl peptidase-4 inhibitor; GLP-1-RA, glucagon-like peptide-1 receptor agonist; MACE, major adverse cardiovascular event; SGLT2i, sodium-glucose cotransporter 2 inhibitor; SU, sulfonylurea and glinides.

Comparative effectiveness was not substantially altered by (1) using the HdPS approach, (2) correcting estimates for IPCW, (3) using a different definition of MACE, or (4) investigating the Sicilian population. Moreover, a tendency in a risk reduction of all-cause death and MACE was observed in patients who received second and third-generation SUs, as compared with those receiving glinides (online supplemental table S3). A protective action of GLP-1-RA and SGLT2i (and, with a lower extent, of DPP-4i) on primary clinical outcomes was observed even when glinides were excluded from the comparison group (online supplemental table S4).

**Figure 1:**
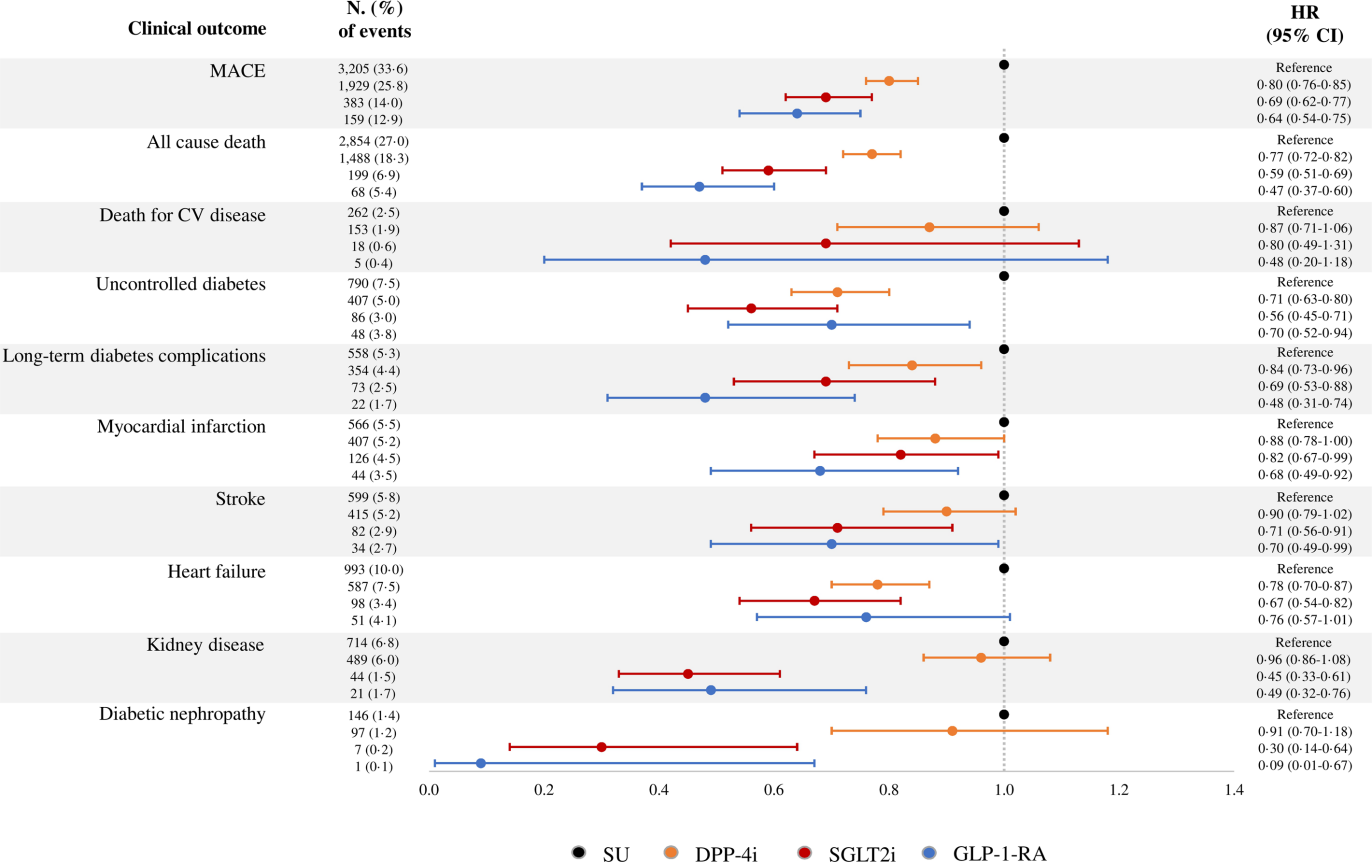
Forest plot of HR and corresponding 95% CI showing the effect of newer second-line agents relative to sulfonylureas and glinides (SU) and the risk of primary and secondary clinical outcomes estimated from the unmatched cohort. CV, cardiovascular; DPP-4i, dipeptidyl peptidase-4 inhibitor; GLP-1-RA, glucagon-like peptide-1 receptor agonist; MACE, major adverse cardiovascular event; SGLT2i, sodium-glucose cotransporter 2 inhibitor.

A forest plot of HR and corresponding 95% CI showed the effect of newer second-line agents relative to SU and the risk of primary and secondary clinical outcomes estimated from the unmatched cohort (figure 1). In addition to confirming the effects of newer agents on primary outcomes, significant reductions in hospital admissions were observed for (1) uncontrolled diabetes and long-term diabetes complications among patients who received all three newer diabetes second-line agents; (2) myocardial infarction, stroke, kidney disease, and diabetic nephropathy among those who received SGLT2i and GLP-1-RA; and (3) heart failure among those who received DPP-4i and SGLT2i. The following were observed compared with patients who received DPP-4i: (1) those on GLP-1-RA had better protection against long-term diabetes complications (p=0.0054), myocardial infarction (p=0.0452), kidney disease (p=0.0011), and diabetic nephropathy (p=0.0108); and (2) those on SGLT2i had better protection against uncontrolled diabetes (p=0.0236), stroke (p=0.0301), kidney disease (p=0.0000), and diabetic nephropathy (p=0.0023). No significant differences in secondary outcomes were observed between patients treated with GLP-1-RA or SGLT2i.

Similar and consistent results were observed when comparing secondary clinical outcomes of DPP-4i, GLP-1-RA and SGLT2i users with second and third-generation SU users (online supplemental figure S2).

### Costs and cost-effectiveness profiles

**Figure 2:**
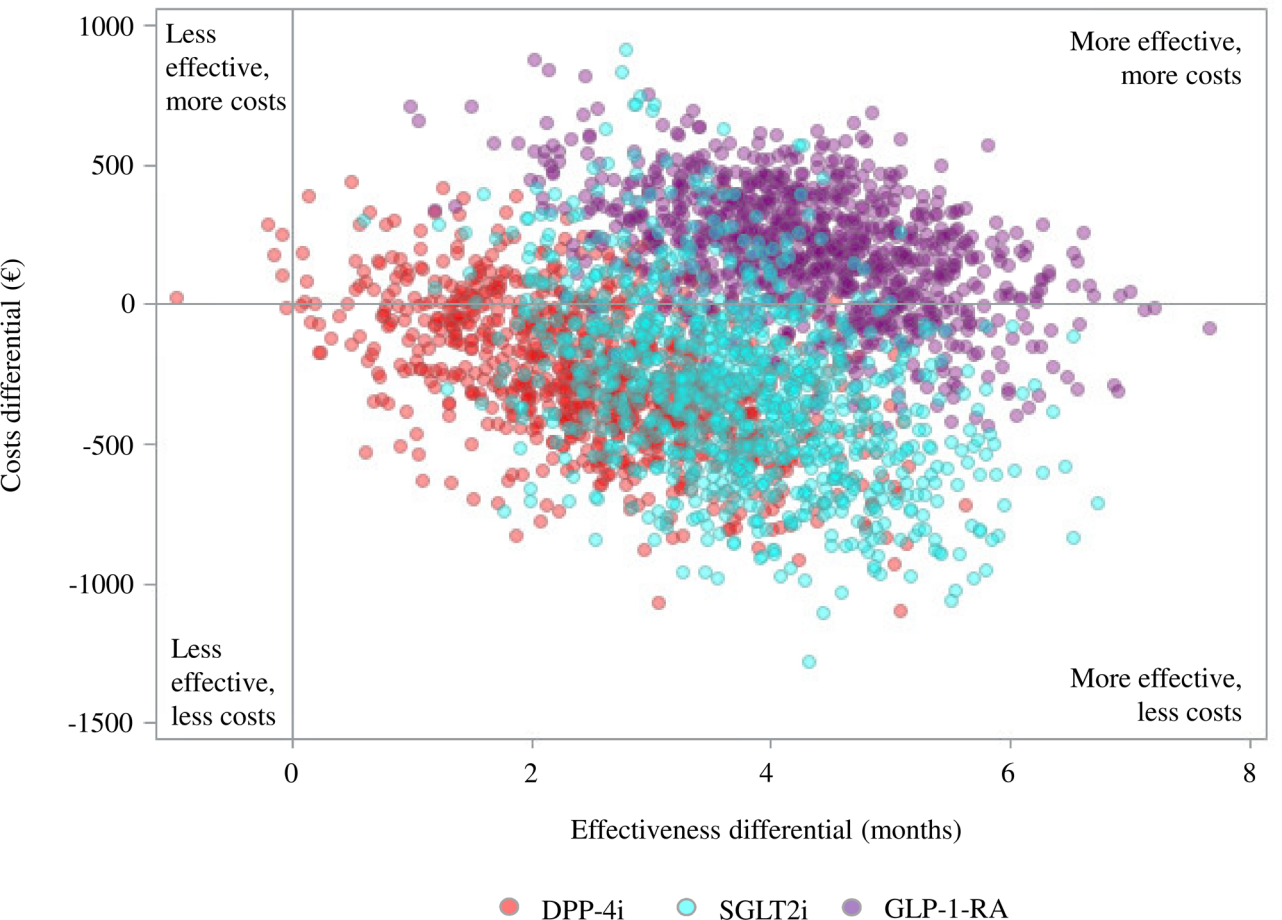
Incremental cost-effectiveness ratio (ICER) scatterplot comparing newer second-line agents relative to sulfonylureas and glinides. DPP-4i, dipeptidyl peptidase-4 inhibitor; GLP-1-RA, glucagon-like peptide-1 receptor agonist; SGLT2i, sodium-glucose cotransporter 2 inhibitor.

Despite the higher cost of newer second-line agents, patients who received either DPP-4i or SGLT2i had reduced average annual healthcare costs of €251 and €282, respectively, compared with those who received SU (table 3). These savings were due to reduced hospitalization costs. Conversely, the high cost of GLP-1-RA was not offset by reduced hospitalization costs for patients who received these drugs. The ICER values indicated an average gain of €96.2 and €75.7 each month free from MACE for patients on DPP-4i and SGLT2i, respectively. In contrast, an average additional cost of €41.8 was incurred for patients on GLP-1-RA (figure 2). The most favorable cost-effectiveness profile based on better effectiveness and saving costs occurred in 83.5% (DPP-4i), 83.6% (SGLT2i), and 18.8% (GLP-1-RA) of the 1000 bootstrap replications.

**Table 3:** Average annual healthcare costs in euros

	SU n=1208	DPP-4i n=1208	SGLT2i n=1208	GLP-1-RA n=1208
Hospitalization	1869	1318	1349	1076
Antidiabetic drugs	467	782	871	1520
Other free-of-charge drugs	652	639	596	679
Outpatient services for diabetes monitoring	34	31	22	30
Other free-of-charge outpatient services	742	743	644	642
Total costs	3764	3513	3482	3947

DPP-4i, dipeptidyl peptidase-4 inhibitor; GLP-1-RA, glucagon-like peptide-1 receptor agonist; SGLT2i, sodium-glucose cotransporter 2 inhibitor; SU, sulfonylurea and glinides.

## Discussion

Based on the observation of a large, unselected population of patients with type 2 diabetes starting second-line therapy after metformin monotherapy failure, this study investigated whether newer diabetes agents such as DPP-4i, SGLT2i, and GLP-1-RA could delay or prevent clinically relevant outcomes and improve cost-effectiveness profiles compared with conventional SU.

The newer diabetes agents were more effective than SU in delaying CV events and death. This effect was observed for all three newer diabetes agents for the composite MACE and all-cause death. Conversely, myocardial infarction, stroke, and kidney outcomes were only improved for patients who received SGLT2i or GLP-1-RA but not for those who received DPP-4i. Furthermore, our study showed that patients treated with SGLT2i or GLP-1-RA had better clinical outcomes than those who received DPP-4i, and these outcomes were significantly better than those who received SU. Consistently with these findings, postmarket randomized controlled trials performed between 2008 and 2020 on newly approved glucose-lowering medications by the US Food and Drug Administration showed favorable effects of GLP-1-RA and SGLT2i agents on CV and kidney outcomes and showed that DPP-4i did not protect against these outcomes.^24^ Similar results were observed in two recent investigations.^25 26^ These findings indicate that GLP-1-RA and/or SGLT2i may be the optimal approaches for treating patients with CV and kidney comorbidities,^27 28^ and that DPP-4i agents may not benefit those with these comorbidities.^29^ Consistent with a recent study in the USA,^29^ patients with CV and kidney disease and those with worse clinical profiles were more likely to receive SU than newer drugs such as GLP-1-RA and SGLT2i. Addressing this treatment/benefit paradox may help reduce the morbidity associated with these conditions.^28^ The recent GRADE(Glycemia Reduction Approaches in Type 2 Diabetes: A Comparative Effectiveness Study) trial compared clinical outcomes of glimepiride, liraglutide and sitagliptin added to metformin-treated patients.^30^ Although it was designed and powered to evaluate metabolic/glycemic outcomes, a significant reduction in the incidence of any adverse event (which included severe hypoglycemia, lactic acidosis, pancreatitis, diabetic ketoacidosis, revascularization, congestive heart failure or cancer) among liraglutide and glimepiride was observed, consistently with our results which showed an incidence reduction in several secondary clinical outcomes. However, in the GRADE study no significant differences in all-cause mortality were observed between treatment arms, likely due to the low statistical power.

We observed that second and third-generation SU users had a better clinical profile and a lower incidence of clinical outcomes than glinide users. This might have overestimated the observed risk reductions in patients treated with DPP-4i, GLP-1-RA and SGLT2i, as compared with SUs. However, when glinides were excluded from the comparison group, consistent results were observed.

Our study showed that roughly €300 every PY should be saved by initiating second-line therapy with DPP-4 or SGLT2i rather than with SU. However, because of its greater protective effects, starting with SGLT2i would provide the best cost-effectiveness profile with €75.7 saved for obtaining a month free from MACE, against €96.2 for initiating with DPP-4. Finally, despite higher effectiveness, starting second-line therapy with GLP-1-RA caused an increased cost of €41.8 every month, free from MACE, due to the high cost of this drug.

Our study has several strengths. First, it was based on a vast, unselected population, which was made possible because the healthcare system in Italy is free or almost free of cost for virtually all citizens. Second, the hospital and outpatient data in the database are accurate because all services claimed by the health providers to obtain reimbursement from the Regional Health Authority are checked, and incorrect reports may have legal consequences. Finally, the consistency of estimates provided by sensitivity analyses supports the robustness of our findings. Moreover, the study was designed to include only patients who started (incident users) a second-line therapy of SU, DPP-4, GLP-1-RA or SGLT2i after metformin. This allowed to avoid prevalent user bias, since all patients were followed up from the first prescription of the drug of interest^31^; immortal time bias, since patients were not susceptible to a period of follow-up during which the study outcomes cannot occur^32^; and time lag bias, which would have occurred if we compared patients not at the same stage of the disease, such as metformin-only (a first-line treatment) users versus second-line treatments.^32^

Our study was subject to the following limitations. First, our study was based on healthcare utilization databases (HUD), which may not always have complete or high-quality data. Second, we can only determine which prescriptions were dispensed at the pharmacy but cannot determine which patients took the prescribed medications. Third, we did not have access to laboratory results, lifestyle parameters, primary healthcare data, or socioeconomic data. Therefore, additional factors may be involved in the choice of therapeutic strategy, such as body mass index, HbA1c levels or renal function. Because patients in worse health were more likely to receive conventional therapies, residual confounding may have affected our results. Specifically, reduced occurrence of clinical outcomes among patients starting second-line therapy with newer diabetes drugs might have been due to better clinical and/or socioeconomic conditions that were not captured using our HUD. We used multiple approaches to minimize the potential for residual confounding, including matching design and validation of the main findings in a very different setting where confounders may have other effects. However, treatment with newer agents may be a surrogate for overall health-seeking behavior in that patients receiving new drugs might also have more regularly followed healthy lifestyle advice and treatment indications.

In conclusion, evidence that newer diabetes drugs are more effective and cost-effective as second-line options following metformin monotherapy for the treatment of type 2 diabetes than classic glucose-lowering agents was shown in our study. DPP-4i was the least effective, and GLP-1 was the most effective; however, GLP-1-RA was the least cost-effective, and SGLT2i was the most cost-effective. Personalizing the choice of newer second-line agents to individual characteristics and risk profiles^33^ may be critical for regulators, clinicians, and patients.

## Supplementary material

10.1136/bmjdrc-2023-003991 online supplemental file 1

## Data Availability

Data are available upon reasonable request.
