# Supplementary material for: Comparing the effectiveness and cost-effectiveness of sulfonylureas and newer diabetes drugs as second-line therapy for patients with type 2 diabetes
Source: BMJ Open Diabetes Res Care. 2024 May 27;12(3):e003991. doi: 10.1136/bmjdrc-2023-003991 (PMC11131106; doi:10.1136/bmjdrc-2023-003991)
Supplement: online supplemental file 1 [file bmjdrc-2023-003991supp001.docx]

**Supplementary Tables S1. Diagnostic ICD-9-CM codes and ATC codes of drugs used in the current study.**

| **Diagnoses** | **ICD-9-CM codes** |
| --- | --- |
| Uncontrolled diabetes | 250.02; 250.12; 250.22; 250.32 |
| Stroke | 430-435 |
| Heart failure | 428; 398.91; 402.01; 402.11; 402.91; 404.01; 404.03;  404.11; 404.13; 404.91; 404.93 |
| Myocardial infarction | 410; 411.0; 412; V45.81; V45.82 |
| Long-term diabetes complications | 250.4; 250.5; 250.6; 250.7; 250.8; 250.9 |
| Kidney diseases | 582; 585; 586; 588; 583.0; 583.1; 583.4; 583.7; 583.8; 584.6 |
| Diabetic nephropathy | 250.40; 250.42 |
| Respiratory diseases | 460-519 |
| Cancer | 140-239 |
| Retinopathy | 362.0; 362.01; 362.02; 362.55; 361; 364.42; 365.63; 369 |
| Depression | 296.2; 296.3; 296.82; 296.90; 298.0; |
| **Drugs** | **ATC codes** |
| Antidiabetic drugs | A10B |
| SGLT2i | A10BK; A10BD15; A10BD16; A10BD20 |
| GLP-1-RA | A10BJ |
| DPP-4i | A10BH; A10BD07; A10BD08; A10BD10; A10BD11; A10BD13; A10BD18; |
| Sulfonylureas | A10BB; A10BC; A10BD01; A10BD02; A10BD04;  A10BD06 |
| Glinides | A10BX02; A10BX03; A10BX08; A10BD14; |
| Metformin | A10BA02 |
| Insulin | A10A |
| Antihypertensive | C02; C03; C07; C08; C09; C10BX03; C10BX09 |
| Antiplatelet | B01AC; C10BX08; C10BX02; C10BX05; C10BX01; N02BA01 |
| Anticoagulant | B01AA; B01AE; B01AF |
| Antidepressant | N06A |
| NSAIDs | M01A |
| Respiratory drugs | R03 |

**Supplementary Table S2.** Baseline characteristics of patients treated with second-line glinides and 2^nd^ or 3^rd^ generation sulfonylureas. Unmatched cohort.

|  | Glinides  N = 2,739 | Second generation sulfonylureas  N = 5,638 | Third generation sulfonylureas  N = 2,200 |
| --- | --- | --- | --- |
| Median follow-up (months) | 60.7 | 67.3 | 71.1 |
| Males | 1472 (53.7) | 3080 (54.6) | 1217 (55.3) |
| Age categories |  |  |  |
| <60 | 210 (7.7) | 1123 (19.9) | 450 (20.5) |
| 60–69 | 522 (19.1) | 1583 (28.1) | 681 (30.9) |
| 70–79 | 1029 (37.6) | 1982 (35.1) | 731 (33.2) |
| ≥80 | 978 (35.7) | 950 (16.9) | 338 (15.4) |
| Duration of treatment with metformin at index date (years) |  |  |  |
| <5 | 765 (27.9) | 1668 (29.6) | 722 (32.8) |
| 5–9 | 1230 (44.9) | 2675 (47.4) | 1029 (46.8) |
| ≥10 | 744 (27.2) | 1295 (23.0) | 449 (20.4) |
| Co-treatments |  |  |  |
| Antihypertensive | 2474 (90.3) | 4577 (81.2) | 1748 (79.4) |
| Antiplatelet | 1338 (48.9) | 1891 (33.5) | 730 (33.2) |
| Anticoagulant | 452 (16.5) | 486 (8.6) | 150 (6.8) |
| Antidepressant | 568 (20.7) | 898 (15.9) | 342 (15.5) |
| Respiratory drugs | 771 (28.2) | 1300 (23.1) | 473 (21.5) |
| NSAIDs | 1017 (37.1) | 2159 (38.3) | 829 (37.7) |
| Comorbidities |  |  |  |
| Stroke | 100 (3.7) | 105 (1.9) | 48 (2.2) |
| Heart failure | 373 (13.6) | 205 (3.6) | 46 (2.1) |
| Myocardial infarction | 133 (4.9) | 134 (2.4) | 41 (1.9) |
| Renal diseases | 222 (8.1) | 55 (1.0) | 12 (0.6) |
| Respiratory diseases | 415 (15.2) | 312 (5.5) | 67 (3.1) |
| Neurological diseases | 17 (0.6) | 19 (0.3) | 8 (0.4) |
| Retinopathy | 1 (0.0) | 8 (0.1) | 4 (0.2) |
| Cancer | 258 (9.4) | 327 (5.8) | 99 (4.5) |
| Depression | 14 (0.5) | 16 (0.3) | 7 (0.3) |
| Multisource comorbidity score |  |  |  |
| Low | 951 (34.7) | 3393 (60.2) | 1378 (62.6) |
| Intermediate | 1333 (48.7) | 1872 (33.2) | 709 (32.2) |
| High | 455 (16.6) | 373 (6.6) | 113 (5.1) |

**Supplementary Table S3.** Association between second-line glinides and 2^nd^ or 3^rd^ generation sulfonylureas and primary clinical outcomes.

|  | Second-line agent | | |  |
| --- | --- | --- | --- | --- |
|  | Glinides | Second generation sulfonylureas | Third generation sulfonylureas | |
| # patients | 2,739 | 5,638 | 2,200 | |
| MACE ^a^ |  |  |  | |
| # (%) of events | 1,024 (46.0) | 1,582 (30.1) | 599 (28.8) | |
| HR (95% CI) | Reference | 0.93 (0.86-1.01) | 0.91 (0.82-1.01) | |
| MACE new ^b^ |  |  |  | |
| # (%) of events | 317 (14.3) | 557 (10.6) | 214 (10.3) | |
| HR (95% CI) | Reference | 0.93 (0.80-1.07) | 0.89 (0.74-1.06) | |
| All-cause death |  |  |  | |
| # (%) of events | 1,137 (41.5) | 1,276 (22.6) | 441 (20.1) | |
| HR (95% CI) | Reference | 0.91 (0.84-0.99) | 0.83 (0.74-0.93) | |

^a^ Myocardial infarction, stroke, heart failure or all-cause deaths

^b^ Myocardial infarction, stoke or CV deaths

**Supplementary Table S4.** Association between second-line therapies and primary clinical outcomes according to the main analysis and selected sensitivity analyses, excluding glinides from the comparison group.

|  | Second-line agent | | | |
| --- | --- | --- | --- | --- |
|  | 2^nd^/3^rd^ generation SU | DPP-4i | SGLT-2i | GLP-1-RA |
| **Main analysis** | | | | |
| # patients | 1,001 | 1,001 | 1,001 | 1,001 |
| MACE^a^ |  |  |  |  |
| # (%) of events | 180 (18.7) | 139 (14.7) | 124 (12.7) | 130 (13.4) |
| HR (95% CI) | Reference | 0.84 (0.65–1.07) | 0.75 (0.58–0.96) | 0.65 (0.51–0.84) |
| All-cause death |  |  |  |  |
| # (%) of events | 107 (10.7) | 83 (8.3) | 57 (5.7) | 54 (5.4) |
| HR (95% CI) | Reference | 0.78 (0.56–1.07) | 0.66 (0.46–0.93) | 0.47 (0.33–0.69) |
| **High dimensional propensity score approach** | | | | |
| # patients | 1,088 | 1,088 | 1,088 | 1,088 |
| MACE^a^ |  |  |  |  |
| # (%) of events | 217 (19.9) | 200 (18.4) | 147 (13.5) | 154 (14.2) |
| HR (95% CI) | Reference | 0.99 (0.80–1.21) | 0.75 (0.60–0.94) | 0.70 (0.56–0.88) |
| All-cause death |  |  |  |  |
| # (%) of events | 120 (11.0) | 96 (8.8) | 72 (6.6) | 57 (5.2) |
| HR (95% CI) | Reference | 0.88 (0.66–1.18) | 0.74 (0.54–1.01) | 0.46 (0.32–0.65) |
| **Probability-of-censoring weights approach** | | | | |
| # patients | 7,838 | 8,125 | 2,893 | 1,272 |
| MACE^a^ |  |  |  |  |
| # (%) of events | 477 (6.1) | 418 (5.1) | 109 (3.8) | 47 (3.7) |
| HR (95% CI) | Reference | 0.71 (0.57-0.83) | 0.67 (0.48-0.94) | 0.67 (0.42-1.06) |
| All-cause death |  |  |  |  |
| # (%) of events | 1038 (13.2) | 829 (10.2) | 97 (3.4) | 40 (3.2) |
| HR (95% CI) | Reference | 0.80 (0.70-0.91) | 0.65 (0.49-0.86) | 0.63 (0.42-0.95) |
| **Different definition of MACE^b^** | | | | |
| # patients | 1,001 | 1,001 | 1,001 | 1,001 |
| MACE^b^ |  |  |  |  |
| # (%) of events | 90 (9.0) | 82 (8.2) | 71 (7.1) | 63 (6.3) |
| HR (95% CI) | Reference | 0.99 (0.72-1.37) | 0.88 (0.63-1.22) | 0.62 (0.44-0.89) |

^a^ Composite outcome including myocardial infarction, stroke, heart failure or all-cause deaths

^b^ Composite outcome including myocardial infarction, stoke or CV deaths

**Supplementary figures.**

**Supplementary Figure S1.** Flow-chart of cohort selection.

**
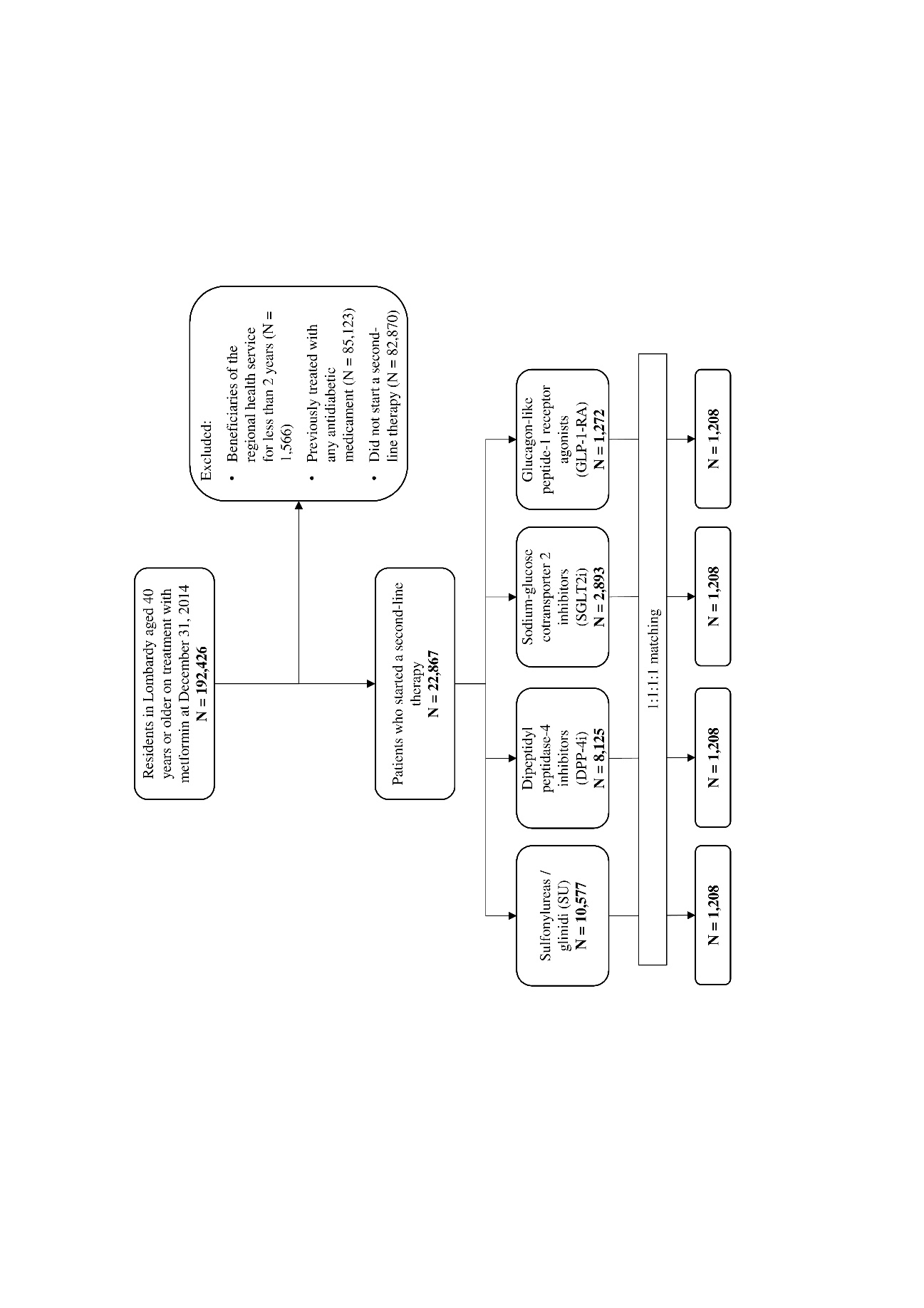
**

**Supplementary Figure S2.** Forest plot of Hazard Ratio (HR) and corresponding 95% Confidence Interval (CI) showing the effect of newer second-line agents relative to 2^nd^/3^rd^ generation sulfonylureas and the risk of primary and secondary clinical outcomes estimated from the unmatched cohort.

**
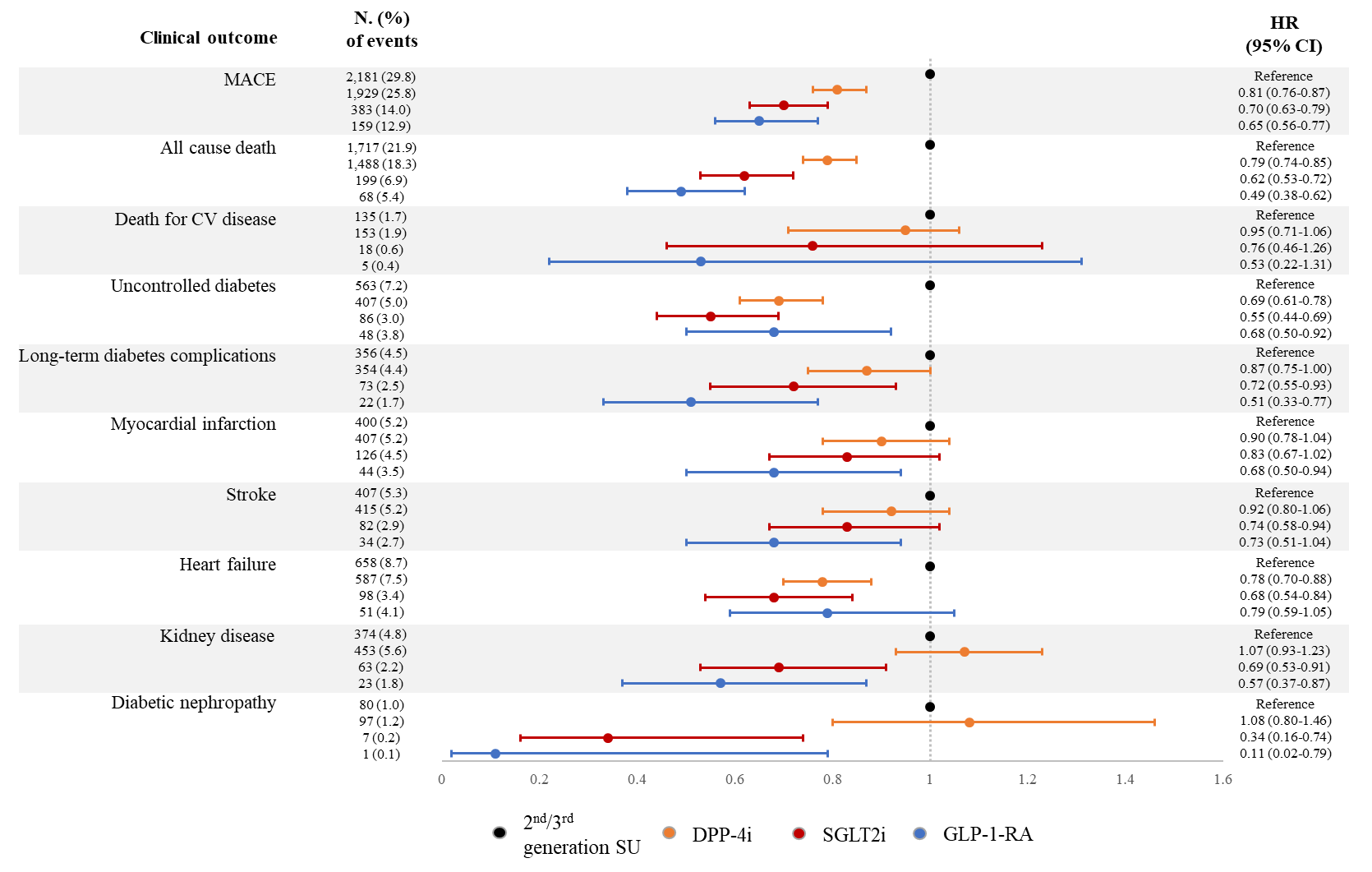
**
